# Stromal or exosome derived lncRNAs regulate stemness in hepatocellular carcinoma

**DOI:** 10.3389/fcell.2026.1957154

**Published:** 2026-09-29

**Authors:** Tianyu Gao, Kai Li, Lin Wang, Xiaomeng Gui, Yuqing Xue, Haojing Li, Meng Qiao, Yaqin Tan, Hong Yang

**Affiliations:** 1 Baotou Medical College, Inner Mongolia University of Science and Technology, Baotou, Inner Mongolia, China; 2 Department of Oncology, Inner Mongolia People’s Hospital, Hohhot, Inner Mongolia, China; 3 Inner Mongolia Medical University, Hohhot, Inner Mongolia, China; 4 Department of Gastrointestinal Oncology, Baotou Tumor Hospital, Baotou, Inner Mongolia, China

**Keywords:** CAFs, exosomal lncRNAs, HCC, stromal cell, TME, tumor cell stemness, tumor molecular mechanisms

## Abstract

**Background:**

Hepatocellular carcinoma (HCC) remains one of the most prevalent malignancies worldwide, with high recurrence rates and chemotherapy resistance posing major clinical challenges. Cancer stem cells (CSCs) are recognized as an important contributors of HCC initiation, metastasis, and recurrence. Within the tumor microenvironment (TME), cancer-associated fibroblasts (CAFs) can maintain CSC properties through intercellular communication. Exosomes serve as essential mediators of this process by delivering long noncoding RNAs (lncRNAs) from various TME cells (including CAFs, MSCs, macrophages, and HCC cells themselves) to recipient HCC cells.

**Methods:**

A literature search of PubMed and Web of Science Core Collection (inception to March 2026) was conducted using key terms including “HCC”, “CAFs”, “MSCs”, “Macrophages”, “Exosome”, “LncRNA”, and “Neoplastic Stem Cells”. Studies were selected based on topic relevance and methodological rigor; those with incomplete or inaccessible data were excluded. The retrieved literature was synthesized to elucidate the mechanisms by which lncRNAs modulate downstream signaling pathways and to evaluate their translational potential as diagnostic biomarkers and therapeutic targets.

**Results:**

This review identifies multiple mechanisms through which stromal or exosome derived lncRNAs regulate HCC cell stemness, including YAP liquid–liquid phase separation, chromatin remodeling via recruitment of SWI/SNF, INO80, NuRD, and BRM/BRG1 complexes, Wnt/β-catenin pathway modulation through protein stabilization and competing endogenous RNA (ceRNA) networks, STAT3 pathway regulation via direct transcriptional repression and transcription factor recruitment, and BMP signaling pathway engagement. Functional classification reveals that these lncRNAs converge on core stemness pathways through complementary transcriptional, posttranscriptional, and protein-level regulatory mechanisms, while integrating signals from the TME.

**Conclusion:**

Stromal or exosome derived lncRNAs constitute a critical regulatory layer in HCC stemness maintenance and hold significant promise as noninvasive diagnostic biomarkers via liquid biopsy. Antisense oligonucleotide (ASO)-based therapeutic strategies targeting the stromal cell–lncRNA–CSC axis represent a promising direction. Nevertheless, systematic characterization of stromal or exosome derived lncRNA species, elucidation of lncRNA sorting and uptake mechanisms, and clinical validation of biomarker and therapeutic applications remain important challenges for future investigation.

## 1 Introduction

Hepatocellular carcinoma (HCC) is the sixth most common malignancy worldwide and the fourth leading cause of cancer-related death, with a 5-year survival rate of less than 20% among patients diagnosed at advanced stages (Li et al., 2026). In recent years, significant progress has been made in the treatment of HCC, including surgical resection, liver transplantation, and targeted therapies. Nevertheless, high recurrence rates and chemotherapy resistance remain major bottlenecks limiting patient prognosis. Accumulating evidence indicates that cancer stem cells (CSCs), also referred to as tumor-initiating cells (TICs), represent a small subpopulation of cells within tumors endowed with self-renewal and multilineage differentiation capabilities. These cells are recognized as one of the root causes of HCC initiation, metastasis, and recurrence (Sakaguchi et al., 2022; Koyama et al., 2020; Chen et al., 2025).

The tumor microenvironment (TME) constitutes the local milieu surrounding tumor cells and comprises tumor cells, immune cells, mesenchymal cells, and other stromal components (Fatima, 2025). Within this context, in-depth investigations into CSCs and cancer-associated fibroblasts (CAFs) have opened new avenues for elucidating the cellular mechanisms underlying tumor progression. In studies pertaining to the reprogramming of cellular stemness, a systematic review of the literature reveals that existing findings remain largely fragmented, with individual studies operating in relative isolation and failing to coalesce into a coherent theoretical framework that enables cross-validation and mutual support. Nevertheless, certain overlaps and commonalities can be identified across these independent studies. Therefore, we attempt to synthesize the mechanisms involved in reprogramming HCC cell stemness and to identify key molecular nodes that may serve as potential targets for future drug development. CAFs, the most abundant stromal cell type within the TME, actively participate in tumor progression by secreting growth factors and cytokines and remodeling the extracellular matrix (Liu et al., 2021; Klingenberg et al., 2017). Exosomes are extracellular vesicles measuring 30–150 nm in diameter that transport nucleic acids, proteins, and lipids, thereby remodeling the behavior of recipient cells (Huo et al., 2017). Long noncoding RNAs (lncRNAs), a class of transcripts exceeding 200 nucleotides in length with limited protein-coding potential, have garnered widespread attention as key regulators in cancer biology (Tran et al., 2025). LncRNAs modulate gene expression at the transcriptional, posttranscriptional, and epigenetic levels, and their aberrant expression is closely associated with multiple aspects of HCC pathogenesis (Lin et al., 2018; Lv et al., 2018). Notably, lncRNAs are enriched within exosomes and can be transferred between cells to transduce oncogenic signals (Huo et al., 2017; Farzaneh et al., 2022). LncRNAs regulate tumor cell stemness across multiple cancer types, including colon, gastric, lung and breast cancers (Gu et al., 2020; Xu et al., 2019; Mao and Liu, 2025; Wang et al., 2025), underscoring their potential as critical modulators of tumor initiation, progression, invasion, and metastasis. Nevertheless, a systematic understanding of how HCC-associated lncRNAs specifically regulate the stemness of HCC cells is lacking.

To comprehensively analyze the mechanisms by which lncRNAs mediate stemness regulation in HCC, with a particular emphasis on recent advances involving stromal or exosome derived lncRNAs exosomal lncRNAs, we conducted a literature search of the PubMed and Web of Science Core Collection databases from inception to March 2026. The search was performed using Boolean operators with the following key terms: “HCC”, “CAFs”, “MSCs”, “Macrophages”, “Exosome”, “LncRNA”, “Neoplastic Stem Cells” and so on, restricted to English-language publications. Retrieved records were first screened by title and abstract, followed by full-text review. Studies were included based on topic relevance and methodological rigor (e.g., clearly described experimental design, adequate sample sizes, and appropriate statistical analysis), while those with incomplete data, inaccessible full texts, or no direct relevance to the review topic were excluded. As this is a narrative review, formal systematic-review methodology was not applied. The retrieved literature was synthesized to elucidate the molecular mechanisms underlying lncRNA function, thereby providing a structural explanation for how these molecules modulate downstream cellular signal transduction pathways.

## 2 Exosomal lncRNAs play critical roles in regulating HCC stemness

### 2.1 Classification and functional diversity of lncRNAs in HCC

The human genome encodes thousands of lncRNAs originating from diverse genomic contexts, including long intergenic noncoding RNAs (lincRNAs), intronic lncRNAs, antisense transcripts, and pseudogene-derived RNAs. In HCC research, bioinformatics analyses have facilitated the identification of numerous aberrantly expressed lncRNAs implicated in tumorigenesis, disease progression, and stemness maintenance (Lv et al., 2018; Wu and Wang, 2017). These lncRNAs exhibit substantial functional heterogeneity, exerting either convergent or antagonistic effects through multiple molecular mechanisms: chromatin remodeling via the recruitment of protein complexes, transcriptional modulation through interactions with transcription factors, posttranscriptional regulation as competing endogenous RNAs (ceRNAs), and control of protein stability and subcellular localization (Zhu et al., 2016a; Wang et al., 2019) (Table 1; Figure 1).

**TABLE 1:** Function summary of HCC stemness-associate lnRNAs.

lncRNA	Source/Localization	Function	Functional overview	Molecular Mechanism	References
NEAT1*	CAF-derived exosomes; HCC nucleus	[+]	Promotes YAP liquid-liquid phase separation enhancing HCC cell stemness; induces autophagy-mediated radioresistance; maintains CD44 expression and CSC properties	a, d	(Sakaguchi et al., 2022; Koyama et al., 2020; Chen et al., 2025)
NEAT1_v1	HCC cells	[+]	Induces autophagy via GABARAP conferring radioresistance; maintains CD44 expression and CSC properties; CD44-independent stemness maintenance	d	(Koyama et al., 2020; Chen et al., 2025)
H19*	Tumor-initiating hepatocytes; macrophage-induced; EpCAM+CD133+ CSCs	[+]	Oncofetal lncRNA maintaining progenitor cell properties; promotes EMT and stem cell transformation via miR-193b/MAPK1 axis; plasma biomarker associated with prognosis	c	(Zhang J. et al., 2019; Rojas et al., 2022)
HOTAIR*	HCC cells	[+]	Activates Bmi-1/P14/P16 signaling via EZH2-mediated miR-218 suppression; downregulates c-Met promoting hybrid EMT phenotype	a	(Topel et al., 2020; Fu et al., 2015)
MALAT1*	HCC cells (classical exosomal marker)	[+]	Regulates stemness-like properties via Wnt/β-catenin axis; knockdown downregulates β-catenin, Stat3, c-Myc, and CD133/CD90	a	(Chang et al., 2020; He et al., 2019)
lncTCF7	CD13^+^CD133+ hepatic CSCs	[+]	Recruits SWI/SNF complex to TCF7 promoter activating Wnt signaling driving self-renewal and tumor propagation	a	(Wang et al., 2015)
lncBRM	Hepatic CSCs	[+]	Binds BRM initiating BRG1/BRM switch activating YAP1 signaling driving self-renewal	a	(Zhu et al., 2016a)
HAND2-AS1	Hepatic CSCs (human-mouse conserved)	[+]	Recruits INO80 complex to BMPR1A promoter activating BMP signaling; ASO targeting synergizes with BMPR1A siRNA for antitumor effects	a	(Wang et al., 2019)
lnc-DILC	HCC tissues and CSCs (downregulated)	[-]	Directly binds IL-6 promoter suppressing transcription; blocks IL-6/STAT3 signaling inhibiting CSC expansion; prognostic marker	b	(Wang et al., 2016)
lncHDAC2	Hepatic CSCs	[+]	Recruits NuRD complex suppressing PTCH1 activating Hedgehog signaling promoting self-renewal and tumor propagation	a	(Wu et al., 2019)
lnc-β-Catm	HCC tumors and hepatic CSCs	[+]	Binds β-catenin and EZH2 promoting β-catenin methylation suppressing ubiquitin-mediated degradation stabilizing β-catenin maintaining Wnt signaling	d	(Zhu et al., 2016b)
DANCR	Stemness-like HCC cells	[+]	Acts as ceRNA sponging miR-199a/214/320a derepressing CTNNB1 promoting stemness features and tumor colonization	c	(Yuan et al., 2016)
lncSox4	Hepatic tumor-initiating cells	[+]	Binds and recruits Stat3 to Sox4 promoter activating Sox4 transcription promoting self-renewal	b	(Chen et al., 2016)
HOXA11-AS	HCC stem cells	[+]	Epigenetically regulates HOXA11 via promoter methylation activating Wnt signaling promoting proliferation, invasion, and self-renewal	a	(Guo et al., 2019)
RUNX1-IT1	HCC cells (downregulated under hypoxia)	[-]	Binds miR-632 as ceRNA promoting GSK-3β suppressing Wnt/β-catenin and stemness; hypoxia-HDAC3 regulates its expression	c	(Sun et al., 2020)
lnc-ROR*	HCC cell-derived exosomes (TGF-β/hypoxia-enriched)	[+]	Enriched in HCC-derived EVs under TGF-β stimulation; transferred to recipient HCC cells via EVs; promotes CD133+ tumor-initiating cell expansion, sphere formation, and chemoresistance	c, d	(Takahashi et al., 2014)
TUG1*	CAF-derived exosomes	[+]	CAF-secreted exosomal TUG1 promotes HCC cell migration, invasion, and glycolysis via miR-524-5p/SIX1 axis; EV characterization by TEM, WB (CD63, CD9, TSG101), confocal uptake; *in vivo* xenograft	c	(Lu et al., 2022)
H19* (exosomal)	CD90^+^ liver CSC-derived exosomes	[+]	CD90^+^ CSC exosomes enriched in H19; promotes angiogenic phenotype and cell adhesion in endothelial cells; EV characterization by DLS, WB (Alix, Tsg101, HSC70), PKH26 uptake; gain/loss of function validated	d	(Conigliaro et al., 2015)
TINCR	HCC tissues and LCSCs	[+]	Binds PTBP1 promoting ATG5 transcription activating autophagy maintaining LCSC self-renewal	b	(Shi et al., 2022)
LINC00680	HCC tissues	[+]	Sponges miR-568 upregulating AKT3 activating mTOR signaling enhancing stemness and 5-FU resistance	c	(Shu et al., 2021)
lncRNA-MUF	MSC-derived	[+]	Binds ANXA2 and acts as miR-34a ceRNA activating Wnt/β-catenin and EMT promoting hepatocarcinogenesis	c, d	(Yan et al., 2017)
DNM3OS	MSC-conditioned medium-induced	[+]	Binds KDM6B reducing TIAM1 promoter H3K27me3 enhancing HCC invasion and metastasis	a	Wang et al. (2021)
ICR	ICAM-1+ CSCs	[+]	Maintains CSC characteristics via RNA duplex formation stabilizing ICAM-1 mRNA promoting portal vein tumor thrombus formation	d	(Guo et al., 2016)
PVT1	Oncofetal lncRNA; HCC cells	[+]	Binds and stabilizes NOP2 protein promoting proliferation and stemness-like properties	d	(Wang et al., 2014)
RAB30-DT	Malignant epithelial cells	[+]	CREB1 transcriptionally activated; binds and stabilizes SRPK1 promoting nuclear localization reshaping alternative splicing driving stemness	b, d	(Si et al., 2025)
PHAROH	HCC cells and embryonic stem cells	[+]	Binds TIAR promoting MYC translation; MYC is a core regulator of stemness	d	(Yu et al., 2021)
LINC00662	HCC cells	[+]	Reduces tumor suppressor gene promoter methylation via MAT1A/SAM axis promoting oncogene activation	a	(Zhong et al., 2022)
n339260	HCC cells	[+]	Promotes vasculogenic mimicry and CSC development; VM is a characteristic function of CSCs	-	(Zhao et al., 2018)
LPAL2	Pseudogene-derived lncRNA (downregulated)	[-]	Suppresses MMP9 expression; downregulation associated with poor prognosis affecting proliferation, invasion, and sphere formation	d	(Lin et al., 2022)
lncCAMTA1	Hepatic CSCs and HCC	[+]	Binds CAMTA1 promoter inducing repressive chromatin structure suppressing CAMTA1 transcription promoting CSC-like properties	a	(Lu et al., 2018)
Lnc34a	HCC tissues	[+]	Recruits DNMT3a and HDAC1 epigenetically suppressing miR-34a activating TGF-β/Smad4 axis promoting bone metastasis	a, c	(Zhang L. et al., 2019)

**[+]** Pro-oncogenic/Pro-stemness; **[-]** Tumor-suppressive/Stemness-inhibitory; * Exosomal marker molecule.

**Molecular Mechanisms:** a. Chromatin remodeling via recruitment of protein complexes; b. Transcriptional regulation through interactions with transcription factors.

c. Post-transcriptional control as competing endogenous RNAs (ceRNAs); d. Regulation of protein stability and localization.

**FIGURE 1:**
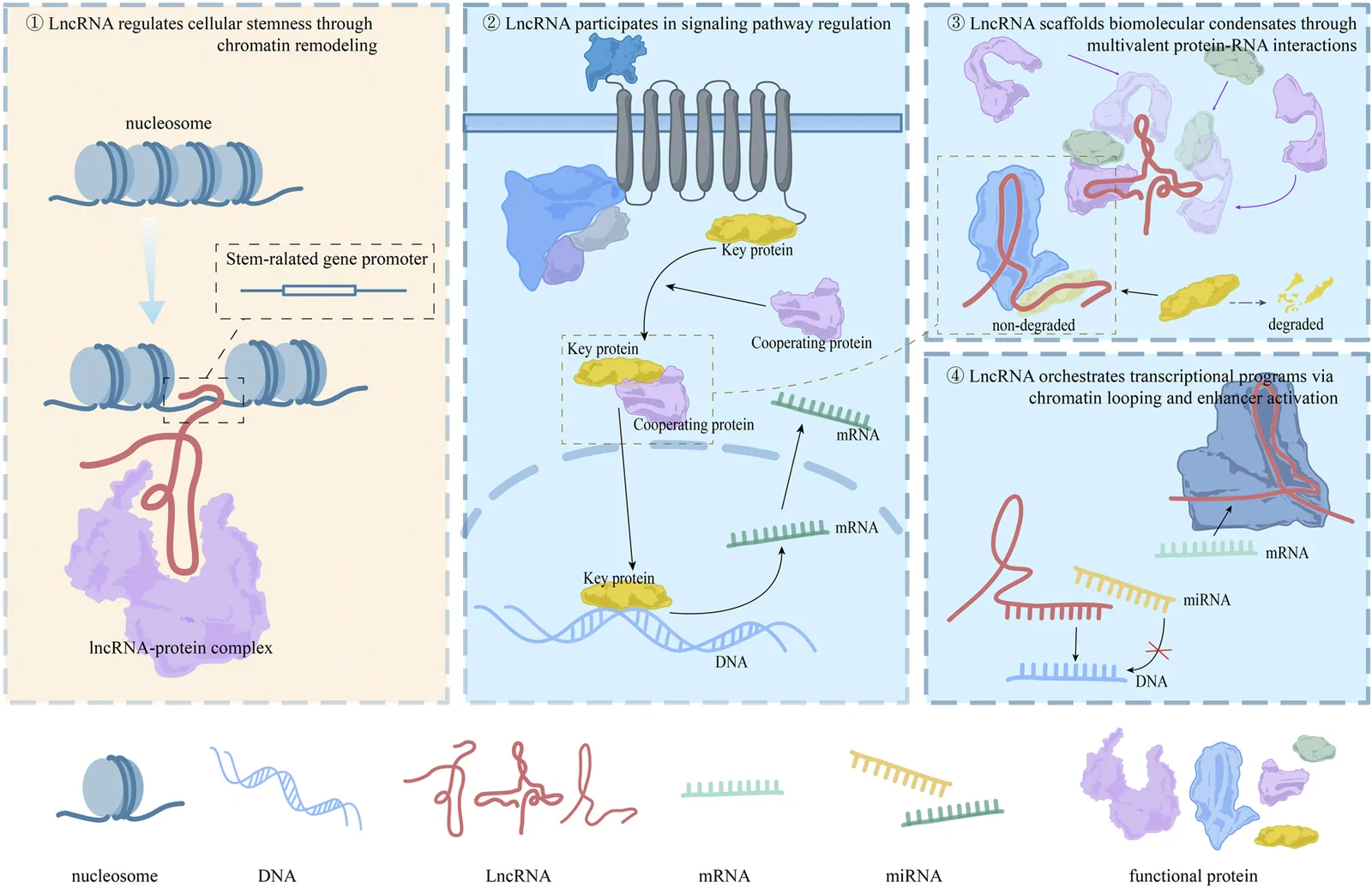
Schematic illustration of the molecular mechanisms by which lncRNAs regulate HCC stemness. (1) LncRNAs recruit chromatin remodeling complexes to stem-related gene promoters, altering nucleosome positioning and activating stemness transcriptional programs. (2) LncRNAs participate in signaling pathway regulation by interacting with key proteins and cooperating proteins, modulating downstream DNA transcription. (3) LncRNAs scaffold biomolecular condensates through multivalent protein-RNA interactions, affecting protein ubiquitination and degradation. (4) LncRNAs orchestrates transcriptional programs via chromatin looping and enhancer activation, regulating mRNA and miRNA expression.

The functional spectrum of HCC stemness-associated lncRNAs reveals substantial diversity in both molecular identity and biological activity. While certain lncRNAs promote stemness maintenance and tumor progression, others exhibit tumor-suppressive properties. For example, lncBRM, HAND2-AS1, and lncTCF7 are highly expressed in hepatic cancer stem cells and are essential for self-renewal and maintenance of the HCC-CSC population (Zhu et al., 2016a; Wang et al., 2019; Wang et al., 2015), whereas lnc-DILC and RUNX1-IT1 suppress CSC expansion and serve as prognostic indicators of favorable outcomes (Wang et al., 2016; Sun et al., 2020). This functional dichotomy underscores the pivotal role of lncRNAs in regulating HCC stemness, thereby identifying promising avenues for investigating the underlying complex regulatory networks. Notably, given the distinct abundance and distribution patterns of exosomes and lncRNAs within the TME, researchers have employed multiple complementary methods to verify the exosomal origin of lncRNAs. According to the MISEV 2018 guidelines (Théry et al., 2018) comprehensive EV characterization requires an integrated approach, including particle analysis, vesicle-marker analysis with appropriate positive and negative controls, morphological analysis, and cargo-association analysis. Several of the studies reviewed herein employed combinations of these techniques, while others relied on more limited characterization approaches, with the specific verification methodologies described in the methodology sections of the original studies.

### 2.2 Exosomes as vectors for lncRNA transmission in the tumor microenvironment

Emerging evidence has established that exosomes are pivotal mediators of intercellular communication within the TME. These extracellular vesicles originate from multivesicular bodies and are released upon fusion with the plasma membrane; their cargo composition is selectively packaged and partially reflects the molecular characteristics of donor cells, with substantial donor-cell-dependent differences in EV composition. Encapsulation within exosomes protects lncRNAs from RNase-mediated degradation, thereby ensuring stable delivery to recipient cells and subsequent regulation of gene expression and cellular phenotypes (Farzaneh et al., 2022; Chang et al., 2020).

In hepatocellular carcinoma, diverse cell populations contribute to the exosomal lncRNA pool, including neoplastic cells, immune constituents, and stromal elements. Mesenchymal stem cells (MSCs) and macrophages transmit lncRNAs to HCC cells, thereby promoting epithelial‒mesenchymal transition (EMT), invasive capacity, and stemness maintenance (Gu et al., 2021; Xu et al., 2022). Notably, the specific regulatory roles of CAF-derived exosomal lncRNAs in HCC stemness have only recently been elucidated, offering refined insights into the intricate intercellular interplay and functional modulation within the HCC TME.

## 3 Acquisition and maintenance of stemness in HCC-CSCs

Tumor cell stemness refers to the capacity for self-renewal, tumor-initiating ability, cellular plasticity, and generation of heterogeneous progeny, and is a critical driver of tumor initiation and progression (Loh and Ma, 2024). In summary, the maintenance of stemness is attributed primarily to aberrant signaling pathway activation, TME remodeling, viral infection-induced alterations, epigenetic modifications, and metabolic adaptation. These interconnected mechanisms form a complex regulatory network that collectively promotes the acquisition and maintenance of stem cell-like properties in HCC cells, thereby contributing to tumor recurrence, metastasis, and drug resistance.

Our previous studies revealed aberrant activation of signaling pathways (e.g., Wnt/β-catenin, PI3K/AKT/mTOR, IL-6/STAT3, and NOTCH) (Chen et al., 2021; Chan et al., 2025; Tsui et al., 2020; Yang et al., 2023), TME remodeling (e.g., Warburg effect-induced hypoxic conditions and inflammatory cell infiltration), and viral infection-induced effects (e.g., direct impact of HBV/HCV proteins on tumor cell DNA) as the most common and predominant mechanisms underlying stemness maintenance in HCC cells (Schulte et al., 2020). Among these, exosomal lncRNAs are involved in pathways such as the Wnt/β-catenin, Hedgehog, and JAK-STAT pathways, as well as epigenetic regulatory mechanisms, including chromatin remodeling, DNA methylation, and histone modifications.

## 4 Molecular mechanisms of lncRNA-mediated regulation of stemness

### 4.1 Exosomal lncRNAs in HCC stemness regulation

#### 4.1.1 CAF-derived exosomal NEAT1 promotes stemness via YAP liquid–liquid phase separation

A study published in 2025 provides direct evidence that CAF-derived exosomal lncRNAs regulate stemness in HCC cells, distinguishing it as one of the few studies in the field to directly demonstrate CAF-to-HCC exosomal transfer. This study identified nuclear-enriched abundant transcript 1 (NEAT1) as a key lncRNA enriched in CAF-derived exosomes that is capable of driving CSC maturation and inducing HCC initiation (Chen et al., 2025).

NEAT1 has two major isoforms: NEAT1_v1 (short isoform, ∼3.7 kb) and NEAT1_v2 (long isoform, ∼23 kb). Significant NEAT1 overexpression in HCC tissues is correlated with poor clinical outcomes. Importantly, high levels of NEAT1 in circulating exosomes are associated with an adverse prognosis, suggesting its potential as a liquid biopsy biomarker. Functional validation has demonstrated that the transfer of NEAT1 from CAFs to HCC cells promotes cancer cell stemness and enhances 3D spheroid formation capacity (Chen et al., 2025).

The function of NEAT1 suggests a molecular mechanism distinct from conventional transcriptional or posttranscriptional regulation. NEAT1 directly binds to the intrinsically disordered region (IDR) of the transcriptional coactivator Yes-associated protein (YAP), as demonstrated by RNA pull-down and RNA immunoprecipitation (RIP) assays (Chen et al., 2025). This interaction promotes YAP liquid–liquid phase separation (LLPS), leading to the formation of biomolecular condensates that enrich the transcriptional machinery and amplify YAP-mediated gene expression programs essential for stemness maintenance, as supported by *in vitro* phase separation assays, fluorescence recovery after photobleaching (FRAP) analysis, and concentration-dependent experiments with CAF-derived EVs (Chen et al., 2025). This finding suggests a mechanism by which CAF-associated exosomal lncRNAs regulate cellular stemness through protein-level modulation.

The NEAT1-YAP-LLPS axis connects microenvironmental signals to intracellular transcriptional programs. YAP, a core effector of the Hippo signaling pathway, is a well-established regulator of CSC properties in HCC (Wu et al., 2022). By promoting YAP phase separation, CAF-derived exosomal NEAT1 effectively amplifies oncogenic signaling in recipient HCC cells, establishing a positive feedback loop that sustains stemness. Clinical observations further support this mechanism, revealing a positive correlation between NEAT1 and Nanog expression in HCC tissues (Chen et al., 2025).

Additional studies have characterized the role of intracellular NEAT1 in maintaining HCC stemness through distinct mechanisms. It should be noted that these studies primarily characterized intracellular NEAT1 functions in HCC cells and complement, rather than directly demonstrate, the CAF-derived exosomal NEAT1 mechanism described above. NEAT1_v1 is required for maintaining the expression of the HCC-CSC marker CD44; its overexpression enhances CSC properties even in CD44-deficient cells, suggesting additional stemness-regulatory mechanisms (Chen et al., 2025). Furthermore, NEAT1_v1 induces autophagy by upregulating gamma-aminobutyric acid receptor-associated protein (GABARAP), a key protein for autophagosome-lysosome fusion, thereby conferring radioresistance in HCC cells (Sakaguchi et al., 2022). Collectively, these findings delineate intracellular NEAT1 functions in HCC stemness regulation, providing complementary insights to the CAF-derived exosomal NEAT1 mechanism.

#### 4.1.2 Lnc-ROR promotes CD133+ tumor-initiating cell expansion via EV-mediated transfer

Lnc-ROR (lncRNA regulator of reprogramming) has been identified as a stress-responsive lncRNA enriched in HCC cell-derived extracellular vesicles under TGF-β stimulation (Takahashi et al., 2014). Takahashi et al. demonstrated that EV-mediated transfer of lnc-ROR to recipient HCC cells increased CD133+ tumor-initiating cell expansion, enhanced sphere formation capacity, and conferred chemoresistance to sorafenib and doxorubicin (Chang et al., 2020). siRNA-mediated knockdown of lnc-ROR attenuated TGF-β-induced CD133+ cell expansion and restored chemotherapy sensitivity, implicating EV lnc-ROR as a functional mediator of cancer stem cell properties and therapeutic resistance in HCC.

#### 4.1.3 CAF-derived exosomal TUG1 promotes HCC migration and glycolysis

TUG1 (taurine upregulated gene 1) represents a CAF-derived exosomal lncRNA with direct relevance to the stromal-HCC communication axis. Increasing evidence suggests that TUG1 is crucial for tumor progression; however, its role in HCC and the underlying mechanisms remained poorly characterized until recently. Lu et al. demonstrated that cancer-associated fibroblasts secreted exosomes that delivered TUG1 to HCC cells, where it promoted migration, invasion, and glycolysis by sponging miR-524-5p and upregulating its downstream target SIX1 (sine oculis homeobox homolog 1) (Lu et al., 2022). The promotive effects of CAF-secreted exosomes were attenuated by silencing of TUG1, confirming that TUG1 is the functional cargo mediating the CAF-to-HCC signaling. Clinically, TUG1 and SIX1 were found to be increased in HCC patients with metastasis, while miR-524-5p was decreased, supporting the *in vivo* relevance of this regulatory axis.

This study provided comprehensive EV characterization consistent with MISEV 2018 recommendations. *In vivo*, silencing of TUG1 suppressed tumor growth and lung metastasis and improved survival in xenograft model mice (Lu et al., 2022). While TUG1 primarily regulates metabolic reprogramming and metastatic capacity rather than stemness directly, its CAF-derived exosomal origin aligns with the stromal communication theme of this review and highlights the diverse functional roles of CAF-derived exosomal lncRNAs in HCC progression.

#### 4.1.4 CD90^+^ CSC-derived exosomal H19 promotes angiogenesis

Exosomal H19 has been demonstrated to mediate communication from CD90^+^ liver cancer stem cells to the tumor microenvironment. Conigliaro et al. showed that CD90^+^ liver CSC-derived exosomes were enriched in H19 lncRNA and promoted angiogenic phenotype and cell-to-cell adhesion in endothelial cells (Conigliaro et al., 2015). EV characterization included dynamic light scattering, Western blot for Alix/Tsg101/HSC70, and PKH26-labeled uptake confirmation. Gain- and loss-of-function experiments validated that H19 plays a functional role in the exosome-mediated angiogenic phenotype (Conigliaro et al., 2015). This study is particularly relevant as it demonstrates that CSC-derived exosomal lncRNAs can remodel the tumor microenvironment, providing additional evidence for the exosomal lncRNA-mediated regulation of HCC progression.

### 4.2 LncRNAs regulate HCC cell stemness via chromatin remodeling and epigenetic mechanisms

In addition to the NEAT1-LLPS mechanism, numerous lncRNAs regulate HCC stemness through chromatin remodeling complexes, representing a major functional class with high therapeutic relevance.

#### 4.2.1 lncTCF7 recruits the SWI/SNF complex

The long noncoding RNA lncTCF7 was among the first HCC stemness-associated lncRNAs identified through transcriptomic microarray analysis of CD13+CD133+ liver CSCs (Wang et al., 2015). Studies have shown that lncTCF7 is highly expressed in both HCC tissues and CSCs and that its loss significantly impairs tumor cell self-renewal and metastatic capacity.

Mechanistically, lncTCF7 recruits the SWI/SNF chromatin remodeling complex to the promoter of TCF7 (T-cell factor 7, a key transcription factor in Wnt signaling) (Wang et al., 2015). This recruitment enhances TCF7 expression, which in turn activates Wnt/β-catenin signaling to drive liver CSC self-renewal. The SWI/SNF complex utilizes ATP hydrolysis to reposition nucleosomes, thereby regulating chromatin accessibility and transcription factor binding. lncTCF7-mediated SWI/SNF recruitment exemplifies a mechanism by which lncRNAs bridge chromatin modifiers to specific genomic loci, ensuring precise control over stemness programs.

This study highlights the fundamental role of lncRNA‒chromatin interactions in CSC regulation. The lncTCF7-TCF7-Wnt axis reveals a mechanism by which lncRNAs amplify proliferative signaling pathways in tumors.

#### 4.2.2 HAND2-AS1 recruits the INO80 complex to promote CSC stemness maintenance and control differentiation

The lncRNA HAND2-AS1 is highly conserved between humans and mice, is highly expressed in liver CSCs, and is essential for self-renewal maintenance and tumor initiation. HAND2-AS1 functions by recruiting the INO80 chromatin remodeling complex to the promoter of BMPR1A (bone morphogenetic protein receptor type 1A) (Wang et al., 2019). INO80 is an ATP-dependent chromatin remodeler that regulates nucleosome positioning and histone variant exchange. By guiding INO80 to the BMPR1A locus, HAND2-AS1 induces BMPR1A expression and activates BMP signaling, a critical pathway involved in both stem cell maintenance and differentiation control.

In addition to its mechanistic role, the therapeutic importance of HAND2-AS1 has been demonstrated in patient-derived xenograft (PDX) HCC models, where antisense oligonucleotides (ASOs) targeting HAND2-AS1 have enhanced antitumor effects with siRNA-mediated BMPR1A knockdown (Wang et al., 2019). Consistent with these findings, hepatocyte-specific knockout of Hand2-as1 or Bmpr1a in mice impaired BMP signaling and suppressed hepatocarcinogenesis. These observations indicate that HAND2-AS1 represents a potential therapeutic target and validate a treatment strategy that suppresses tumor growth by targeting both a lncRNA and its downstream pathway to inhibit cancer cell stemness. However, clinical translation of ASO-based HAND2-AS1 targeting faces several challenges, including delivery efficiency to HCC cells *in vivo*, potential off-target effects and toxicity, and the need for validation in human studies. These limitations should be addressed in future preclinical and clinical investigations.

#### 4.2.3 lncHDAC2 promotes cellular stemness remodeling via recruitment of the NuRD complex and activation of hedgehog signaling

Zhang et al. demonstrated that lncHDAC2 is highly expressed in liver CSCs and regulates cellular self-renewal by activating Hedgehog signaling through chromatin remodeling coupled with proliferative and differentiation signals (Wu et al., 2019). Notably, this pathway is fundamental to both embryonic development and cancer stemness remodeling.

Mechanistically, lncHDAC2 recruits the NuRD (nucleosome remodeling and deacetylation) complex to the promoter of PTCH1 (Patched 1, the primary receptor for Hedgehog ligands) (Wu et al., 2019). NuRD-mediated chromatin modification represses PTCH1 expression, thereby derepressing Smoothened (Smo) and activating downstream Hedgehog target genes. Importantly, the Smo inhibitor cyclopamine impaired liver CSC self-renewal and suppressed tumor propagation, confirming the therapeutic relevance of the lncHDAC2-Hedgehog axis.

The study further revealed that HDAC2 expression levels are positively correlated with HCC severity, whereas PTCH1 levels are negatively correlated with tumor progression. Therefore, targeting lncHDAC2 or its downstream effectors may benefit HCC patients with activated Hedgehog signaling. These findings also suggest that lncHDAC2 could serve as a prognostic biomarker in HCC and represents a potential target for drug development.

#### 4.2.4 LncBRM-mediated BRM/BRG1 switching and YAP activation are critical steps in CSC self-renewal

Research on LncBRM has revealed mechanistic and functional convergence with the NEAT1-YAP pathway, further underscoring the central role of YAP signaling in lncRNA-mediated stemness regulation. High expression of LncBRM in liver CSCs indicates that LncBRM is required for stemness maintenance and hepatocarcinogenesis (Zhu et al., 2016a).

In liver CSCs, LncBRM binds to BRM (Brahma, a catalytic subunit of the SWI/SNF complex), initiating BRM/BRG1 switching (Zhu et al., 2016a). This switch promotes the formation of BRG1-incorporated BAF complexes, thereby activating YAP1 signaling. The expression levels of LncBRM and YAP1 target genes are positively correlated with HCC severity, suggesting their potential utility as potential combinatorial biomarkers.

Of particular interest is the mechanistic relationship between LncBRM and NEAT1. Whereas LncBRM activates YAP1 through chromatin remodeling-mediated transcriptional upregulation, NEAT1 further amplifies YAP function through protein phase separation (Koyama et al., 2020; Zhu et al., 2016a). These distinct mechanisms suggest multilayered regulation of YAP activity by lncRNAs. Combined targeting of LncBRM and NEAT1 represents a potential therapeutic strategy warranting future investigation.

### 4.3 LncRNAs regulate the Wnt/β-catenin pathway via distinct mechanisms

Among the signaling pathways extensively investigated to date, the Wnt/β-catenin pathway represents the axis most frequently targeted by HCC stemness-promoting lncRNAs Li and Zhu, 2020. In addition to lncTCF7-mediated chromatin remodeling, multiple lncRNAs employ distinct molecular strategies to activate this pathway.

#### 4.3.1 Lnc-β-Catm stabilizes the β-catenin protein via methylation

A study on lnc-β-Catm revealed a novel mechanism of lncRNA-mediated protein modification. This lncRNA is highly expressed in HCC tumors and liver CSCs, whereas loss of lnc-β-Catm impairs CSC self-renewal and tumor propagation (Zhu et al., 2016b). Mechanistically, lnc-β-Catm, β-catenin, and the histone methyltransferase EZH2 form a ternary complex that promotes β-catenin methylation (Zhu et al., 2016b). Methylated β-catenin resists ubiquitination and proteasomal degradation, thereby increasing protein stability and sustaining Wnt/β-catenin signaling.

These findings establish that lncRNAs can regulate proteins at the posttranslational level, extending beyond their well-known roles in transcriptional and posttranscriptional regulation. The identification of the lnc-β-Catm-EZH2-β-catenin axis suggests the presence of druggable targets within this pathway. Indeed, small-molecule inhibitors of EZH2 methyltransferase activity have been proposed as potential agents capable of disrupting this oncogenic circuit (Zhu et al., 2016b).

#### 4.3.2 DANCR functions as a ceRNA to reduce β-catenin expression

Fu et al. (Yuan et al., 2016) demonstrated that DANCR (differentiation antagonizing nonprotein coding RNA) acts as a ceRNA during Wnt pathway activation, thereby blocking miRNA-mediated suppression of cellular stemness (Yuan et al., 2016). DANCR is overexpressed in HCC-CSCs and serves as a prognostic marker. Unlike lncRNAs, which act directly on chromatin or proteins, DANCR functions as a molecular sponge for miR-199a, miR-214, and miR-320a, all of which target CTNNB1 (the mRNA encoding β-catenin) (Yuan et al., 2016).

By sequestering these tumor-suppressive miRNAs, DANCR enhances CTNNB1 expression, leading to enhanced Wnt/β-catenin signaling and increased stemness characteristics. This mechanism highlights the complex interplay between lncRNAs and miRNAs in posttranscriptional gene regulation.

#### 4.3.3 Epigenetic regulation by MALAT1

Metastasis-associated lung adenocarcinoma transcript 1 (MALAT1) is one of the most comprehensively studied long noncoding RNAs and regulates HCC stemness through the Wnt/β-catenin signaling pathway (He et al., 2019). Knockdown of MALAT1 downregulates β-catenin, Stat3, c-Myc, and stemness markers, including CD133 and CD90, while also reducing ALDH-1 activity and tumor sphere formation efficiency.

The MALAT1–Wnt axis represents a therapeutically targetable loop. Pharmacological disruption of MALAT1 expression or its interaction with β-catenin may simultaneously suppress both stemness and metastasis in HCC (He et al., 2019). Given the well-established presence of MALAT1 in exosomes derived from various cancer types, investigating its transfer and crosstalk between CAFs and HCC cells represents a promising direction for future research.

### 4.4 Inflammatory signaling and the STAT3 pathway

Crosstalk between inflammation and stemness is increasingly recognized as a driver of HCC progression. Long noncoding RNAs may serve as key mediators connecting inflammatory signals with stemness programs.

#### 4.4.1 Direct transcriptional repression by lnc-DILC

Lnc-DILC (lncRNA downregulated in liver cancer stem cells) is one of the few lncRNAs identified to date that exhibits stemness-suppressive functions (Wang et al., 2016). Lnc-DILC is expressed at low levels in HCC tissues and CSCs, and its loss enhances HCC-CSC expansion and promotes tumorigenesis.

Lnc-DILC can also directly bind to the IL-6 promoter region and inhibit IL-6 transcription (Wang et al., 2016). This direct lncRNA‒DNA interaction suppresses autocrine IL‒6/STAT3 signaling—a pathway essential for CSC maintenance. The ability of lncRNAs to directly bind genomic DNA and regulate transcription has expanded our understanding of the functional diversity of these molecules.

Lnc-DILC levels correlate with IL-6 expression as well as with EpCAM and CD24 levels, and its predictive value for early recurrence and patient survival has been validated (Wang et al., 2016). Reduced lnc-DILC expression may serve as a prognostic marker, whereas therapeutic strategies aimed at restoring lnc-DILC expression or function may suppress inflammation-driven tumor cell stemness.

Given that IL-6 is a critical factor for CAF activation and maintenance, the lnc-DILC–IL-6–STAT3 axis suggests a plausible hypothesis: CAF-derived exosomal lncRNAs may increase IL-6/STAT3 signaling in recipient HCC cells—either by suppressing lnc-DILC or by activating alternative mechanisms—thereby promoting stemness.

#### 4.4.2 Recruitment of transcription factors by lncSox4

Studies have identified an alternative mechanism by which lncSox4 activates the STAT3 pathway through transcriptional regulation (Chen et al., 2016). This lncRNA is highly expressed in TICs and is essential for their self-renewal. LncSox4 physically interacts with STAT3 and recruits it to the Sox4 promoter, thereby initiating Sox4 transcription (Chen et al., 2016). Sox4, a member of the SRY-related HMG-box (Sox) family of transcription factors, functions as a core regulator of stemness. The lncSox4–STAT3–Sox4 regulatory circuit establishes a positive feedback mechanism that sustains TIC properties, and both lncSox4 and Sox4 expression levels correlate with HCC development and clinical severity (Chen et al., 2016).

### 4.5 BMP signaling and developmental pathways

Although the Wnt and STAT3 pathways dominate stemness regulatory functions, BMP signaling represents an equally important but mechanistically distinct regulatory axis. As described above, HAND2-AS1 activates BMP signaling in the context of chromatin remodeling through INO80-mediated upregulation of BMPR1A (Wang et al., 2019). Whereas BMP signaling typically promotes differentiation and suppresses proliferation in various contexts, appropriate levels of BMP signaling appear to be required for stemness maintenance in liver CSCs, potentially by fine-tuning the balance between self-renewal and differentiation (Wang et al., 2019). Notably, the context-dependent effects of BMP signaling on HCC stemness warrant further investigation, as therapeutic strategies that modulate cellular stemness through this pathway may exert opposing effects across distinct tumor cell subpopulations.

## 5 Functional classification and cross-talk of lncRNA regulatory networks

### 5.1 Convergence on core stemness pathways

Analysis and synthesis of existing studies revealed that, despite the remarkable diversity of lncRNA species and types, they converge significantly onto a limited set of core signaling pathways. The Wnt/β-catenin pathway has emerged as the most frequently targeted axis, with at least four distinct lncRNAs (lncTCF7, lnc-β-Catm, DANCR, and MALAT1) employing different molecular mechanisms to achieve pathway activation (Xu et al., 2019; Chang et al., 2020; Chen et al., 2016; Ye et al., 2020). This convergent evolution suggests that Wnt signaling represents a critical node in liver CSC maintenance and that tumors employ multiple lncRNA-driven regulatory strategies to ensure the robustness of pathway signaling.

Similarly, YAP signaling is activated through both chromatin-mediated (lncBRM) and phase separation-mediated (NEAT1) mechanisms, indicating that Hippo pathway effectors serve as key integrators of microenvironmental signals and intrinsic stemness signals (Zhong et al., 2022). The functional redundancy between these mechanisms may explain the drug resistance observed when individual pathway components are targeted, although this proposed hypothesis requires future experimental validation.

### 5.2 Complementary mechanisms: transcriptional, posttranscriptional, and protein-level regulation

A summary of HCC-associated lncRNA functions reveals the major levels of gene expression regulation. Transcriptional control is achieved through direct DNA binding (e.g., lnc-DILC at the IL-6 promoter) (Wang et al., 2016) as well as the recruitment of chromatin remodeling complexes (e.g., lncTCF7-SWI/SNF, HAND2-AS1-INO80, lncHDAC2-NuRD, and lncBRM-BAF) (Zhu et al., 2016a; Wang et al., 2019; Wang et al., 2015; Wu et al., 2019). Posttranscriptional regulation operates primarily through the ceRNA mechanism, wherein lncRNA sponging of tumor-suppressive miRNAs derepresses oncogenes (e.g., DANCR-miR-199a/214/320a-CTNNB1) (Yuan et al., 2016). Protein-level regulation includes control of posttranslational modifications (e.g., lnc-β-Catm-EZH2-mediated β-catenin methylation), modulation of protein stability (e.g., lncRNA-MUF-ANXA2), and emerging mechanisms such as liquid–liquid phase separation (NEAT1-YAP) (Sakaguchi et al., 2022; Koyama et al., 2020; Chen et al., 2025). This multitiered regulatory architecture provides robustness to cellular stemness programmes and suggests that effective therapeutic intervention in HCC may require simultaneous targeting of multiple regulatory nodes.

To further illustrate the evidence landscape of these lncRNAs, we provide a visual summary classifying them into four evidence tiers based on their EV/exosomal relevance, ranging from direct exosomal transfer (Tier I) to intracellular studies requiring further validation of exosomal involvement (Tier IV) (Figure 2).

**FIGURE 2:**
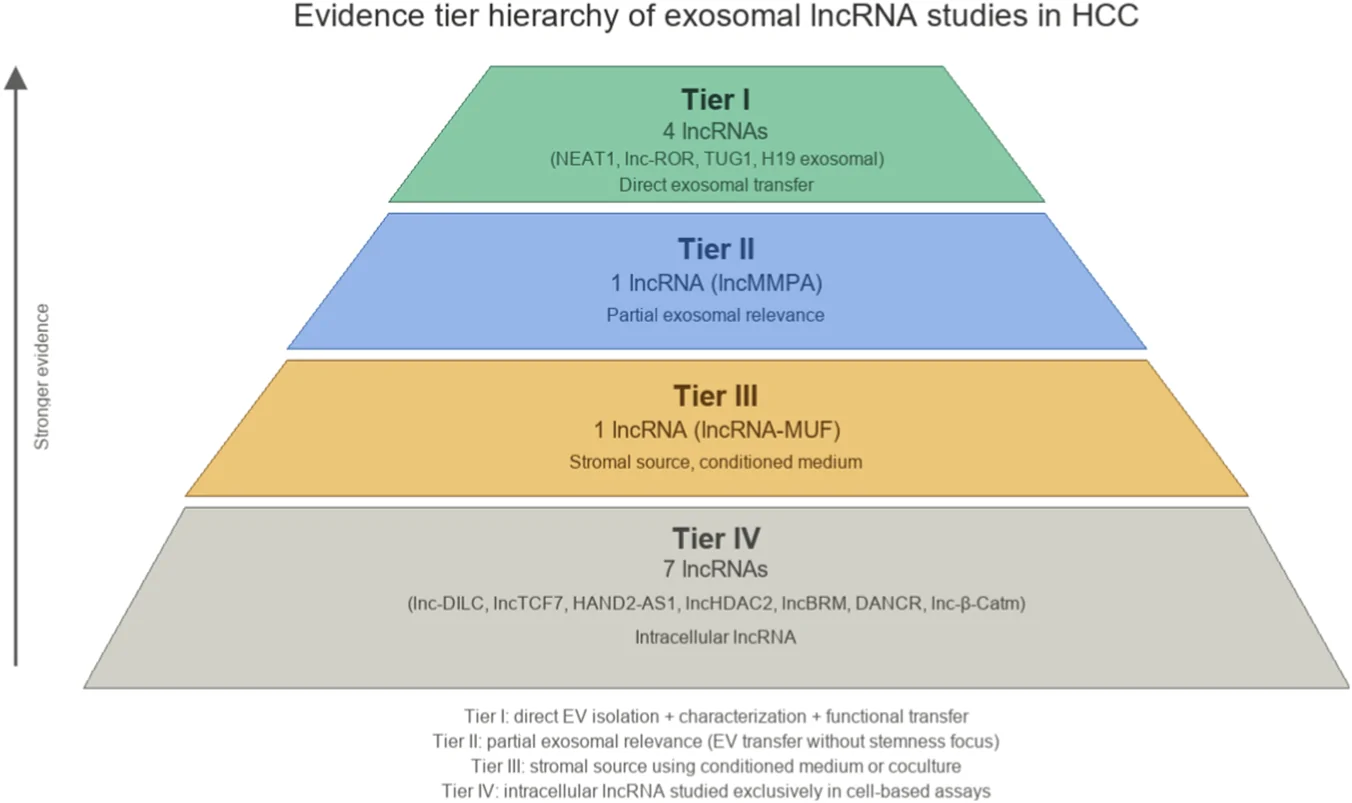
Evidence tier hierarchy of exosomal lncRNA studies in HCC.

Additionally, although the title focuses on stromal or exosome derived lncRNAs, Tier IV lncRNAs are included to provide a comprehensive overview of the stemness regulatory network, as their mechanisms complement those of stromal or exosomal lncRNAs.

### 5.3 Integration of signals from the tumor microenvironment by lncRNAs

Multiple studies have provided indirect support for microenvironment–lncRNA–stemness crosstalk. For example, TGF-β, a classic cytokine that drives CAF activation, regulates H19 expression through the TGF-β/Sox2 axis in tumor-initiating hepatocytes (Zhang J. et al., 2019). Conversely, H19 maintains progenitor cell properties and is highly expressed in CSCs (Rojas et al., 2022). The TGF-β-H19-Sox2 loop thus connects CAF activation to stemness maintenance, although whether this involves exosomal transfer remains to be determined.

Macrophage-induced upregulation of H19 promotes HCC aggressiveness via the miR-193b/MAPK1 axis, demonstrating lncRNA-mediated communication between immune cells and HCC cells (Ye et al., 2020). Similarly, HCC-MSCs induce lncRNA-MUF expression in HCC cells via coculture-mediated paracrine signaling, where lncRNA-MUF activates Wnt/β-catenin signaling and EMT through ANXA2 binding and miR-34a sponging (Yan et al., 2017). Whether EV-mediated transfer of lncRNA-MUF occurs awaits further investigation, as the coculture system allows passage of both EVs and soluble factors. MSCs also induce DNM3OS expression via the KDM6B/TIAM1 axis to increase invasion and metastasis (Wang et al., 2021).

These findings provide credible evidence for signal transmission among stromal cells, HCC cells, and lncRNAs, establishing that play a key role in mediating the phenotypic remodeling of HCC cells within the TME. Nevertheless, comprehensive systematic investigations into the mechanisms governing lncRNA packaging into exosomes and their functional impact on recipient cell stemness are lacking.

## 6 Conclusion

Research on the mechanisms by which HCC-associated exosomal lncRNAs regulate the stemness of HCC cells holds significant translational potential and represents a rapidly advancing field. Current evidence indicates that lncRNAs employ diverse molecular mechanisms—including chromatin remodeling, liquid–liquid phase separation, and signaling pathway modulation—to maintain liver CSC properties. The landmark discovery that CAF-derived exosomal NEAT1 promotes stemness through YAP phase separation provides direct proof-of-concept for microenvironmental lncRNA-mediated stemness regulation and has opened a new paradigm for understanding lncRNA function (Sakaguchi et al., 2022).

This review synthesizes the roles and pathways of individual lncRNAs from independent studies into a comprehensive and systematic regulatory network, aiming to provide researchers with precise directions and innovative insights for future exploration in this field. The convergence of multiple lncRNAs with core pathways (Wnt/β-catenin, YAP, and STAT3) suggests that targeting these pathways, either alone or in combination with lncRNAs that function as upstream inhibitors, may effectively suppress tumor cell stemness.

Systematic characterization of the species and functions of CAF-derived exosomal lncRNAs, elucidation of the mechanisms governing lncRNA sorting and uptake, and clinical validation of lncRNAs as biomarkers and therapeutic targets underscore that important mechanistic questions remain to be addressed in this field. With advances in single-cell technologies, molecular docking, and computational modeling, more comprehensive and integrated investigations into the regulation of tumor cell stemness by exosomal lncRNAs are anticipated, ultimately aiming to uncover the mechanisms underlying the CAF–lncRNA–CSC axis and thereby improve the prognosis of HCC patients.

## 7 Discussion

Synthesis of the studies reviewed herein reveals that lncRNAs present substantial potential for application in the diagnosis and treatment of HCC. Liquid biopsy has attracted considerable attention because of its noninvasive nature and capacity for dynamic monitoring, and circulating lncRNAs possess significant potential as diagnostic biomarkers. The oncofoetal lncRNA H19 is reactivated in HCC, and its plasma levels correlate with BCLC stage, decrease following treatment, and increase upon recurrence, suggesting its utility for therapeutic monitoring and early detection. NEAT1 in circulating EVs is associated with poor prognosis and enables real-time monitoring of tumor microenvironment communication. Conversely, low levels of the tumor suppressor lnc-DILC predict early recurrence and may help identify patients at high risk of relapse.

With respect to therapeutic applications, the ability of lncRNAs to block or compete with miRNAs highlights antisense oligonucleotide (ASO)-based strategies as promising future approaches for cancer therapy. The observed synergistic effect of HAND2-AS1 ASO combined with BMPR1A knockdown, together with studies demonstrating that H19 ASO reduces CSC self-renewal capacity, validates the feasibility of this approach. Furthermore, small-molecule inhibitors targeting lncRNA‒protein interactions have shown considerable promise; for example, compounds that disrupt NEAT1–YAP liquid‒liquid phase separation may suppress stemness without affecting normal cellular functions. Combined targeting of multiple lncRNAs or their downstream pathways may represent an important and effective future therapeutic strategy.

Despite these advances, several knowledge gaps and limitations remain, including systematic characterization of stromal or exosome derived lncRNA species and functions, elucidation of the mechanisms governing lncRNA sorting and uptake into exosomes, and clinical validation of lncRNAs as biomarkers and therapeutic targets. Additionally, the *in vivo* timing and quantitative features of exosomal transfer await further investigation. As single-cell sequencing, multiomics integration, and machine learning technologies have matured and become more widely adopted, it may become possible to define stromal cell or even TME cell subtypes on the basis of exosomal lncRNA profiles, thereby enabling deeper exploration of tumor initiation and progression mechanisms. Moreover, in-depth investigations into how lncRNAs regulate biomolecular condensates and how exosomal lncRNAs reprogram HCC cell metabolism may open new avenues for nongenetic, targeted pharmacological interventions in HCC.

Finally, we acknowledge the inherent limitations of narrative reviews. As this work did not follow a formal systematic search methodology, the literature selection reflects the authors’ judgment based on relevance and methodological rigor, which may introduce inherent selection bias.
